# Cardiac Magnetic Resonance in Pulmonary Hypertension—an Update

**DOI:** 10.1007/s12410-020-09550-2

**Published:** 2020-11-07

**Authors:** Samer Alabed, Pankaj Garg, Christopher S. Johns, Faisal Alandejani, Yousef Shahin, Krit Dwivedi, Hamza Zafar, James M Wild, David G Kiely, Andrew J Swift

**Affiliations:** 1 Department of Infection, Immunity and Cardiovascular Disease, University of Sheffield, Glossop Road, Sheffield, S10 2JF UK; 2 Department of Clinical Radiology, Sheffield Teaching Hospitals, Sheffield, UK; 3 INSIGNEO, Institute for In Silico Medicine, University of Sheffield, Sheffield, UK; 4 Sheffield Pulmonary Vascular Disease Unit, Royal Hallamshire Hospital, Sheffield, UK

**Keywords:** Pulmonary hypertension, Cardiac MRI, CMR

## Abstract

**Purpose of Review:**

This article reviews advances over the past 3 years in cardiac magnetic resonance (CMR) imaging in pulmonary hypertension (PH). We aim to bring the reader up-to-date with CMR applications in diagnosis, prognosis, 4D flow, strain analysis, T_1_ mapping, machine learning and ongoing research.

**Recent Findings:**

CMR volumetric and functional metrics are now established as valuable prognostic markers in PH. This imaging modality is increasingly used to assess treatment response and improves risk stratification when incorporated into PH risk scores. Emerging techniques such as myocardial T_1_ mapping may play a role in the follow-up of selected patients. Myocardial strain may be used as an early marker for right and left ventricular dysfunction and a predictor for mortality. Machine learning has offered a glimpse into future possibilities. Ongoing research of new PH therapies is increasingly using CMR as a clinical endpoint.

**Summary:**

The last 3 years have seen several large studies establishing CMR as a valuable diagnostic and prognostic tool in patients with PH, with CMR increasingly considered as an endpoint in clinical trials of PH therapies. Machine learning approaches to improve automation and accuracy of CMR metrics and identify imaging features of PH is an area of active research interest with promising clinical utility.

## Introduction

Pulmonary hypertension (PH) is a heterogeneous group of diseases that cause an elevated pulmonary artery pressure [1, 2]. Chronic heart and lung diseases are the underlying causes of most PH cases in the Western world [3] while living at high-altitude and schistosomiasis infection are more common in resource-limited countries [4]. Treatable causes include chronic thromboembolic disease (CTEPH) and pulmonary arterial hypertension (PAH) [5].

PH was historically considered as a rare, difficult to diagnose and untreatable disease [6]. However, our understanding of the epidemiology, diagnosis and treatment of PH has changed over the past two decades. Chronic comorbid cardiac and pulmonary diseases causing PH, such as systemic hypertension and COPD, are increasingly common in an ageing population [7–9]. Recent population-based studies suggest that the prevalence of PH has increased by almost 30% over the last 30 years [3]. PH is now estimated to have a prevalence of 1% of the world’s population and might be the fourth most prevalent cardiovascular disease [6, 8].

The management of PH relies on ascertaining the diagnosis, discerning the underlying cause of PH, assessing disease severity and monitoring response to treatment [1, 2]. The role of cardiac magnetic resonance (CMR) in each of these key aspects of PH management has been extensively studied and is increasingly established in clinical practice.

This article reviews significant advances over the past 3 years in CMR imaging in PH. We build on the previous articles by Zitzman et al. and Swift et al. to bring the reader up-to-date on the current developments in CMR applications in PH [10, 11]. We aim to cover the latest articles about diagnosis, prognosis, 4D flow, strain analysis, T_1_ mapping, machine learning and interesting ongoing research.

## Diagnosis

PH is diagnosed by right heart catheter (RHC). The haemodynamic criteria for a diagnosis of PH from international guidelines are an elevated mean pulmonary artery pressure (mPAP) of ≥ 25 mmHg and pulmonary vascular resistance (PVR) of ≥ 3 Wood units [5]. A new mPAP threshold of > 20 mmHg has recently been proposed as a more accurate criterion, being two standard deviations above the normal threshold [5].

In a breathless patient, PH is increasingly suggested on common investigations such as chest radiography, echocardiogram or computer tomography [1, 12]. New advances in noninvasive imaging techniques have helped to establish the diagnosis of PH sooner in the course of the disease [13, 14]. Cardiac MRI has been used to estimate mPAP to provide diagnosis through non-invasive means. A recent study developed regression models to predict mPAP based on cardiac MRI [15]. A cohort of 600 patients who had both CMR and RHC was retrospectively included. The cohort was divided in half, the first half to derive the regression model and the second half to validate its performance. CMR parameters included in the linear regression model were the interventricular septum angle (IVS), ventricular mass index (VMI) and black blood slow flow. The model had a sensitivity of 93% and specificity 79% of detecting PH, allowing for an accurate non-invasive diagnosis. The model highly correlated with mPAP and had good interobserver reproducibility. The authors also recently updated their model in line with the new suggested mPAP threshold of > 20 mmHg [16]. The same group also developed a diagnostic and prognostic CMR model for patients with PH secondary to chronic obstructive pulmonary disease (COPD) [17]. Pulmonary artery (PA) indices of the diastolic area and relative area change in addition to the IVS and VMI were included in the COPD model. This model had a 92% sensitivity and 80% specificity in detecting PH in COPD patients. PA systolic and diastolic areas also had a high diagnostic accuracy in detecting PH in patients with interstitial lung disease [18]. CMR can be used as a one-stop study to provide functional, aetiological and prognostic information [19]. CMR showed a good correlation with RHC parameters and had high sensitivity and specificity in identifying the underlying causes of PH [19].

CMR-guided RHC is a recent technique that allows performing RHC in a CMR suite. It combines the benefits of radiation-free CMR information to haemodynamics in a single sitting. CMR-guided RHC has a rare failure rate and an acceptable procedure time that is comparable to a standard CMR study [20–22].

## Prognosis and Therapy Response

PH is a chronic, progressive and mostly incurable disease with high morbidity and mortality. A new diagnosis of PH increases the risk of death at 1 year by sevenfold [3]. Mortality is attributed to right heart failure resulting from the increased afterload secondary to elevated pulmonary arterial pressures [2]. PAH prognosis, however, has significantly improved with the advancement of treatment, and the median survival has increased from 3 to 7 years over the last 20 years [23, 24].

The ESC/ERS guidelines describe the prognostic factors including imaging parameters in PAH [2]. Large right atrial size and the presence of pericardial effusion on echocardiogram or CMR imaging feature as prognostic markers in the ESC/ERS traffic light system representing low, intermediate and high risk. There is a wider range of prognostic factors, including the right ventricular metrics that are considered crucially important in prognostication. Further work to determine the most potent CMR prognostic factors and their incremental value in prognostic equations is required.

The most recent statement on imaging and PH from the Pulmonary Vascular Research Institute recommends cardiac MRI to monitor right ventricular (RV) function [1]. CMR can be used at follow-up to assess disease progression and treatment response and provide prognostic information.

A recent systematic review, including 1,938 patients, has shown that CMR is a powerful predictor of clinical worsening and mortality in PH [25••]. In particular, worse RV function and larger RV volume are associated with a worse outcome. A large study in 2017 assessed CMR prognostic features in 576 patients [26••]. The study has shown that RV end-systolic volume and pulmonary artery (PA) relative area change have incremental prognostic value over clinical parameters. Pulmonary artery stiffness assessed by a low relative area change and distensibility is associated with a more severe PH and a higher risk of mortality [26–28]. The prognostic features in patients with connective tissue disease (CTD) were shown to be different to other PAH subgroups. In CTD patients, metrics such as ventricular-vascular coupling (Ees/Ea) and RV mass appear to be more significant than function and volume [26••, 29–31]. New therapies have shown to improve RV contractility and reduce RV mass in CTD PAH, and CMR can play an important role in assessing their treatment response [32, 33].

Cardiac MRI assessment of the interventricular septal (IVS) angle helps to differentiate pre- and postcapillary PH from isolated postcapillary PH. An increased angle of ≥ 160° is associated with pre- and postcapillary PH and with a poorer prognosis [34]. In addition, increased trabeculation at the marginal IVS is associated with severe PH, reduced RV ejection fraction (RVEF) and exercise tolerance [35, 36].

A large study has set the prognostic thresholds for CMR indices [37••]. This study assessed the added value of CMR to the validated prognostic calculators such as the Registry to Evaluate Early and Long-Term PAH Disease Management (REVEAL) and the modified French Pulmonary Hypertension Registry (FPHR). The age- and sex-adjusted RV end-systolic volume index improved prognostication when combined with a risk score. The prognostic thresholds will serve as an important guide for cardiac MRI risk stratification in PAH. Notably, one of the most significant predictors for a worse outcome in IPAH was a background of even minor or mild parenchymal lung abnormality. A background of mild fibrosis or emphysema was associated with a 5-year survival of 22% compared to 78% in IPAH without any lung disease [17, 38]. IPAH with lung disease has, therefore, been suggested to be a separate phenotype of PAH [39].

## Myocardial Strain Analysis

**Fig. 1:**
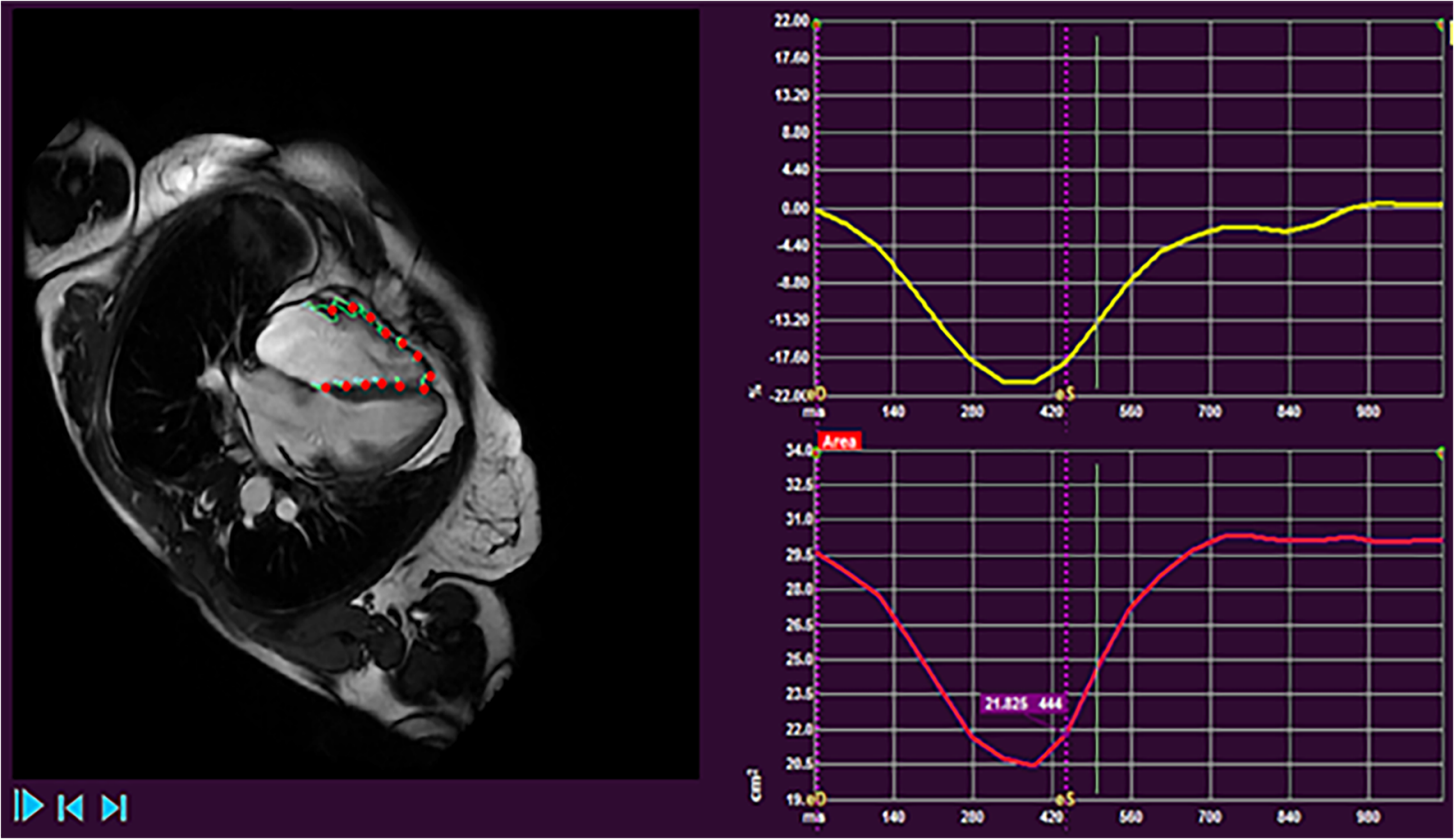
Images from right ventricular strain analysis in a patient with PH

Strain analysis is an established CMR technique for the quantification of myocardial deformation and assessment of wall motion [40]. Feature tracking is one method of strain analysis which follows cardiac borders throughout the cardiac cycle on cine images (Fig. 1). Strain analysis in CMR uses similar assumptions to speckle tracking on echocardiogram with good agreement between the two modalities [41, 42].

Biventricular strain is significantly impaired in PH and could assist in the early detection of right and left heart dysfunction [43–45]. Besides, feature tracking technology has been used to predict outcome in patients with PH. A reduced RV circumferential and longitudinal strain rates were associated with an impaired RVEF and a significant predictor of mortality [46]. The same holds true for the left ventricle (LV) where reduced LV circumferential and longitudinal strain rates in precapillary PH are associated with severely impaired RVEF and a higher risk of death [47]. Impaired right atrial (RA) strain and phasic function are a marker of disease severity. Reduced RA strain is associated with decompensated RV function and stiffness [48, 49]. The advancement of fully automated myocardial strain analysis is likely to push its role in future research in the diagnosis and prognosis of PH [50].

## Myocardial Late Gadolinium Enhancement

Late gadolinium enhancement (LGE) is a CMR technique to identify the areas of myocardial fibrosis. Gadolinium has paramagnetic properties that shorten the myocardial T_1_ times. The T_1_ shortening is proportional to the concentration of gadolinium in the extracellular space. Gadolinium enhancement in the normal myocardium clears out early. However, its clearance is restricted in necrotic tissue due to the expansion of the extracellular space and damage to the cellular membranes of the myocytes [51].

LGE is associated with poor outcome and increased mortality in cardiomyopathies [52]. However, a study assessing LGE in 124 PH patients found that LGE did not predict mortality [53]. This finding confirms the results of two previous studies that suggested no added prognostic information from LGE in PH when added to other CMR parameters [54, 55]. Therefore, LGE in PH and particularly at the RV insertion points or IVS appears to be a consequence of increased mechanical stress and RV remodelling and not a sign of RV decompensation.

## Myocardial T_1_ and Extracellular Volume Mapping

Native myocardial T_1_ and extracellular volume (ECV) mapping are novel biomarkers used in several cardiovascular disorders to aid diagnostic, prognostic and therapeutic decision-making [56]. Myocardial T_1_ mapping is a pixel-by-pixel representation of the longitudinal relaxation times (T_1_) within a tissue [57]. T_1_ values provide surrogate tissue characterisation data that are measured on a standardised scale [58]. Assessing T_1_ post-gadolinium can be used to estimate ECV [56, 59]. The ECV is calculated by subtracting the T_1_ values of the myocardium and blood pool pre- and post-contrast, corrected for the haematocrit level [56]. Elevated T_1_ mapping values and ECV can indicate areas of oedema and fibrosis in the myocardium [60, 61]. Several recent studies have looked into the clinical application of T_1_ mapping and ECV in PH [62–67]. T_1_ times are elevated in PH and in particular at the RV insertion points and are associated with an increased intraventricular septal angle and LV eccentricity [62•, 65]. Increased T_1_ values are therefore thought to be related to RV dilatation and the resultant shift of the septum towards the LV. The diagnostic application in PH, however, remains limited. Although T_1_ looked promising for differentiating between healthy volunteers and PH, the differences were much smaller in patients without a PH diagnosis in a clinical setting [62]. A raised T_1_ value in PH is weakly correlated to RVEF [62•, 64, 68]. However, T_1_ times did not predict mortality in a large cohort of PH patients [62]. An elevated ECV in PH patients with heart failure and preserved ejection fraction was associated with RV dilatation, stiffness and reduced RV strain and therefore might play a role as a marker for RV remodelling [67].

CMR also plays a role in CTEPH treatment response assessment [69, 70]. Septal myocardial T_1_ mapping is elevated in CTEPH patients [63] and reduces after treatment with balloon pulmonary angioplasty [63, 71]. T_1_ mapping may, therefore, be utilised in CTEPH therapy monitoring.

## Pulmonary MR Angiography and Perfusion

MR angiography (MRA) has a high spatial and low temporal resolution that allows for the assessment of the pulmonary vasculature. Perfusion MRI, on the other hand, has a low spatial and a high temporal resolution that enables evaluation of the capillary level tissue perfusion which makes it suitable in the clinical assessment of CTEPH [72].

The diagnostic accuracy of dynamic contrast-enhanced perfusion MRI in the diagnosis of CTEPH was shown to be comparable to computed tomography pulmonary angiography (CTPA) and perfusion single-photon emission tomography (SPECT) [73]. Perfusion MRI identified all cases of CTEPH and had comparable sensitivity and specificity to the other modalities. Recent studies have shown that ventilation and perfusion changes in CTEPH can be interrogated using phase-resolved functional lung MRI without the need for contrast agents [74]. Perfusion MRI is likely to play an essential role in the diagnostic pathway in centres that already perform CMR for CTEPH patients.

## 4D Flow

Four-dimensional flow (4D flow) is an emerging MRI technology that offers to circumvent issues with standard ultrasound imaging in PH. Not only 4D flow allows 3D visualisation of vascular flow, it also allows to make an accurate assessment of transvalvular or intra-cavity flow [75]. In the setting of PH, 4D flow has been used to assess the haemodynamic changes in the pulmonary circulation. Abnormal flow patterns in the main pulmonary artery (MPA), namely vortex formation, have been associated with PH [76, 77]. The presence and ‘persistence time’ of the vortex in the MPA are linearly associated with mPAP [78, 79] and can be used to estimate mPAP. Another physiological vascular parameter characterised with 4D flow is MPA wall shear stress (WSS), which has an impact on vascular remodelling [80]. 4D flow-derived MPA WSS appears to be reduced in patients with PH [80, 81]. Also, in patients with PH who have poor acoustic windows for echocardiography, 4D flow can provide reliable quantification of tricuspid regurgitation [82, 83]. 4D flow could also provide clinically relevant RV diastolic assessment in PH [84, 85]. To summarise, 4D flow MRI can provide several complementary diagnostic information in the assessment of patients presenting with suspected PH. Future studies need to evaluate the incremental role of 4D flow MRI assessment in patients with PH.

## Machine Learning

Machine learning (ML) uses algorithms to recognise patterns in example data to make predictive decisions in unprecedented data [86]. ML can classify the data based on the differentiating patterns it has learnt [87, 88].

ML is likely to play an important role in PH [89]. Recent approaches include automated segmentation [90, 91], biventricular 3D model creation [92], computational models and decision tree analysis [93], diagnosis [94••] and prognostication [95••, 96].

ML has been used to analyse cardiac motion and predict mortality based on reduced ventricular contraction [95••]. The ML model was shown to improve outcome prediction compared to conventional CMR measurements alone.

**Fig. 2:**
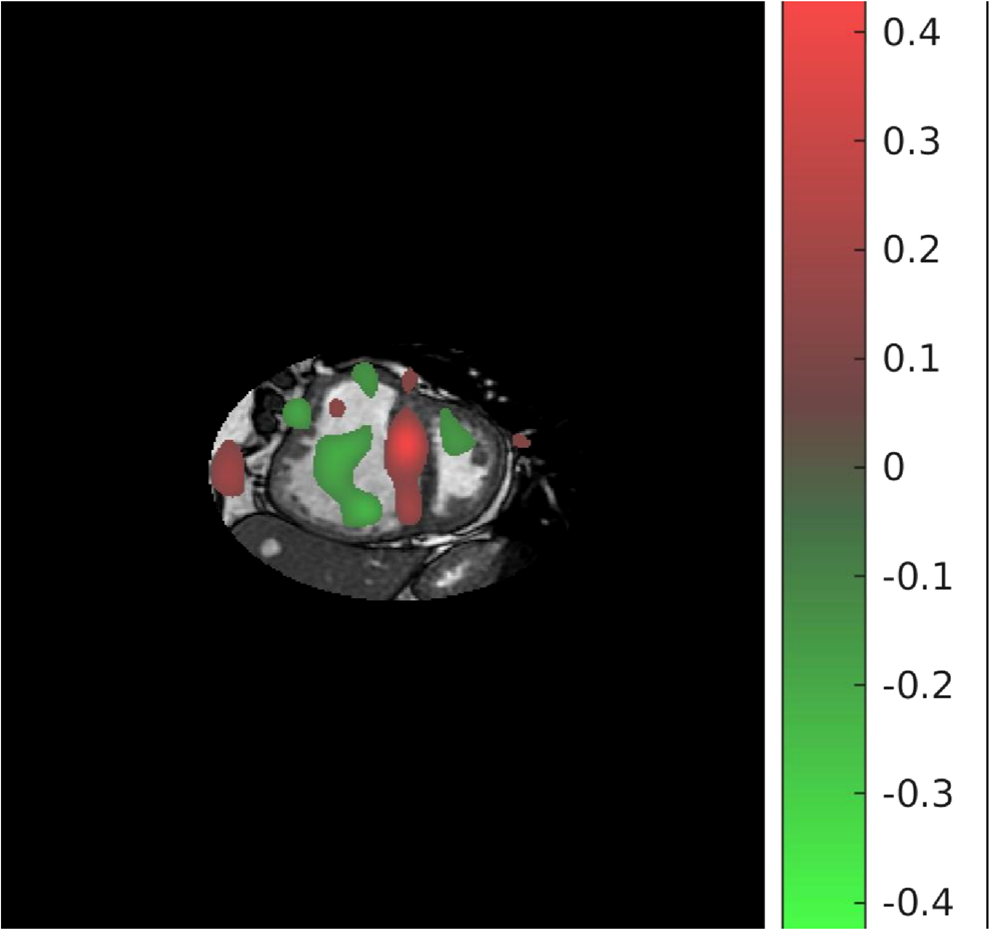
Machine learning feature map. Features compatible with PH are in red and non-PH features are in green

ML was used to identify diagnostic features on CMR and classify them into PH or no PH [94••]. The discriminating features were mapped onto CMR voxel space and were shown as a visual overlay on the 4 chamber and short-axis images (Fig. 2). Interestingly, this approach does not require segmentation of the cardiac chambers, allowing for faster processing and reduced segmentation-induced error. This study gives a glimpse into the future of PH assessment that allows for rapid and accurate CMR diagnoses. An exciting development would be to ML methods utilised in predicting prognosis and treatment response in PH.

## Ongoing Research

### Repeatability of CMR Measurements

The RESPIRE study aims to assess the reproducibility of CMR measurements at follow-up. It will also compare the repeatability of CMR to other endpoints such as walking and blood tests. This study would help establish the evidence of the usefulness of CMR as a monitoring tool and its sensitivity to change [97].

### CMR as Clinical Trial Endpoint

The REPAIR study is the first study to have MRI as a co-primary endpoint [98••]. Four ongoing randomised controlled trials, assessing beta-blockers, spironolactone, CXA-10 and dehydroepiandrosterone, have defined CMR parameters as an endpoint to evaluate treatment response [99–102]. A single-arm study of treprostinil in PH has defined the change in RV structure and function, on CMR compared to an echocardiogram, as the primary treatment response outcome [103].

### Follow-up CMR Assessment

A prospective study is aiming to recruit 180 incident cases of PAH. Participants will have CMR and RHC at baseline and 6- and 24-month follow-up. The aim is to determine poor prognostic markers before decompensation occurs. This research would be valuable in early risk stratification as current studies include patients with more advanced stages of the disease [104].

### CTEPH Diagnosis and Screening

CHANGE-MRI is a large European multicentre study that aims to compare dynamic contrast-enhanced MRI compared to VQ-SPECT in people with suspected CTEPH. This study is anticipated to set the standard for MRI in the diagnostic algorithm for CTEPH [105].

## Conclusions

The last 3 years have seen several large studies examining the clinical utility of CMR in patients with PH. Evidence confirms the potential for CMR to provide diagnostic and prognostic information that can guide clinical practice. CMR has been utilised in clinical trials to detect the impact of PH therapies and is increasingly proposed as a trial endpoint. Machine learning approaches to improve automation and accuracy of CMR metrics and identify imaging features of PH shows potential and is likely to improve the clinical utility of CMR imaging.
